# Editorial: Suicidality and self-injury behaviours across the lifespan in mental and substance use disorders

**DOI:** 10.3389/fpsyt.2025.1639200

**Published:** 2025-07-15

**Authors:** Ruta Karaliuniene, Ramdas Ransing, Laura Orsolini

**Affiliations:** ^1^ Rhein-Mosel-Fachklinik Clinic, Landeskrankenhaus Andernach, Academic Hospital at Johannes Gutenberg, University Mainz, Andernach, Germany; ^2^ Department of Psychiatry, Clinical Neurosciences, and Addiction Medicine, All India Institute of Medical Sciences (AIIMS), Guwahati, Assam, India; ^3^ Unit of Clinical Psychiatry, Department of Neurosciences/DIMSC, Polytechnic University of Marche, Ancona, Italy

**Keywords:** suicidality, nonsuicidal self injury, nonsuicidal self-injurious behavior, mental health, substance use disorders

Suicidality and self-injury continue to represent significant challenges worldwide, particularly among individuals affected by mental health and substance use disorders. These behaviours are complex and multifaceted, influenced by an interplay of biological, psychological, and social factors that evolve across developmental stages and cultural contexts. The collection of articles presented in this *Frontiers in Psychiatry* Research Topic provides timely and valuable insights into these critical phenomena, exploring the underlying mechanisms, risk factors, and potential avenues for intervention.

A prominent theme emerging from this Research Topic is the nuanced relationship between non-suicidal self-injury (NSSI) and suicidal behaviour. While these behaviours are related, they present distinct clinical features and trajectories, requiring careful differentiation and targeted approaches for prevention and treatment.

For example, a population-based study from South Korea by Kim et al. highlighted the association between smoking status and suicidal ideation, planning, and attempts. This research underscores the importance of integrating behavioural health factors into suicide prevention strategies and the need to address modifiable lifestyle risks in public health initiatives.

Adolescence was identified as a particularly vulnerable period in several contributions. Zhu et al. developed a predictive model to identify adolescents at elevated risk for NSSI within six months following psychiatric hospitalisation, emphasising the critical window for early intervention. Complementing this, Xu et al. demonstrated through a longitudinal analysis that NSSI can both precede and result from suicidal ideation among youths, revealing a reciprocal relationship that challenges traditional linear models of risk. These findings highlight the importance of continuous monitoring and individualised support in clinical practice.

Advances in neuroimaging have enhanced our understanding of the biological underpinnings of suicidality. Tymofiyeva et al. focused on the resting-state functional connectivity of the putamen in depressed adolescents with a history of suicide attempts, identifying potential biomarkers that could inform future personalised treatment approaches. In parallel, Orsolini et al. investigated social cognitive deficits, particularly impairments in Theory of Mind, in youths transitioning from NSSI to suicide attempts. Their study suggests that difficulties in understanding others’ mental states may contribute to the progression towards suicidal behaviour.

The role of digital environments in shaping self-injurious behaviour is becoming increasingly recognised. Orsolini et al. explored how online interactions may influence NSSI among young people, highlighting both the risks and opportunities inherent in social media use. Their findings call for the development of innovative prevention strategies that engage youth within digital contexts.

From a diagnostic perspective, two articles critically assessed the DSM-5 criteria for suicidal behaviour and NSSI disorders. Oliogu and Ruocco conducted a comprehensive review of the clinical utility, pathophysiology, and treatment options associated with suicidal behaviour disorder, while Dierickx et al. compared patterns of NSSI engagement and severity among emerging adults. These contributions are vital as the field seeks consensus on diagnostic boundaries and subsequent clinical management.

Cultural and social factors also play a significant role in suicidality and self-injury. Meisler et al. examined NSSI and mental health among female Arab minority students, revealing how identity conflict and acculturation stress shape these behaviours. Their work underscores the need for culturally sensitive interventions that are tailored to diverse populations.

Finally, Pató et al. focused on an often-overlooked group - prisoners, investigating suicidal behaviour through the lens of behavioural addiction. Their study broadens our understanding of the risk factors and mechanisms associated with suicidality in incarcerated populations.

Overall, these studies clearly illustrate the multifaceted nature of suicidality and self-injury, which is shaped by developmental, neurobiological, psychosocial, and cultural influences. All of these studies emphasise the need for an integrated and multidisciplinary approach from the assessment phase onwards, along with a tailored diagnostic and treatment approach adapted to different age groups and target populations.

This Research Topic not only advances knowledge but also identifies important gaps for future inquiry. Longitudinal studies to clarify causality, the refinement of neurobiological markers, and the development of culturally attuned, evidence-based prevention and treatment programmes should be prioritised.

As the global burden of suicide and self-injury persists, especially among individuals with mental health and substance use disorders, this collection offers a valuable framework for clinicians, researchers, and policymakers seeking to reduce harm and save lives throughout the lifespan.

